# Multimodularity of a GH10 Xylanase Found in the Termite Gut Metagenome

**DOI:** 10.1128/AEM.01714-20

**Published:** 2021-01-15

**Authors:** Haiyang Wu, Eleni Ioannou, Bernard Henrissat, Cédric Y. Montanier, Sophie Bozonnet, Michael J. O’Donohue, Claire Dumon

**Affiliations:** a TBI, Université de Toulouse, CNRS, INRAE, INSA, Toulouse, France; b Institute of Biological Environmental and Rural Sciences, Aberystwyth University, Aberystwyth, United Kingdom; c CNRS UMR 7257, Aix-Marseille University, Marseille, France; d INRAE, USC 1408 AFMB, Marseille, France; e Department of Biological Sciences, King Abdulaziz University, Jeddah, Saudi Arabia; University of Manchester

**Keywords:** termite gut, lignocellulose, glycoside hydrolase, carbohydrate-binding module, xylanase, PUL, GH10, CBM4, protein domain insertion, functional genomics

## Abstract

The functional screening of a *Pseudacanthotermes militaris* termite gut metagenomic library revealed an array of xylan-degrading enzymes, including *P. militaris* 25 (*Pm*25), a multimodular glycoside hydrolase family 10 (GH10). Sequence analysis showed details of the unusual domain organization of this enzyme. It consists of one catalytic domain, which is intercalated by two carbohydrate binding modules (CBMs) from family 4. The genes upstream of the genes encoding *Pm*25 are *susC*-*susD*-*unk*, suggesting *Pm*25 is a Xyn10C-like enzyme belonging to a polysaccharide utilization locus. The majority of Xyn10C-like enzymes shared the same interrupted domain architecture and were vastly distributed in different xylan utilization loci found in gut *Bacteroidetes*, indicating the importance of this enzyme in glycan acquisition for gut microbiota. To understand its unusual multimodularity and the possible role of the CBMs, a detailed characterization of the full-length *Pm*25 and truncated variants was performed. Results revealed that the GH10 catalytic module is specific toward the hydrolysis of xylan. Ligand binding results indicate that the GH10 module and the CBMs act independently, whereas the tandem CBM4s act synergistically with each other and improve enzymatic activity when assayed on insoluble polysaccharides. In addition, we show that the UNK protein upstream of *Pm*25 is able to bind arabinoxylan. Altogether, these findings contribute to a better understanding of the potential role of Xyn10C-like proteins in xylan utilization systems of gut bacteria.

**IMPORTANCE** Xylan is the major hemicellulosic polysaccharide in cereals and contributes to the recalcitrance of the plant cell wall toward degradation. Members of the *Bacteroidetes*, one of the main phyla in rumen and human gut microbiota, have been shown to encode polysaccharide utilization loci dedicated to the degradation of xylan. Here, we present the biochemical characterization of a xylanase encoded by a *Bacteroidetes* strain isolated from the termite gut metagenome. This xylanase is a multimodular enzyme, the sequence of which is interrupted by the insertion of two CBMs from family 4. Our results show that this enzyme resembles homologues that were shown to be important for xylan degradation in rumen or human diet and show that the CBM insertion in the middle of the sequence seems to be a common feature in xylan utilization systems. This study shed light on our understanding of xylan degradation and plant cell wall deconstruction, which can be applied to several applications in food, feed, and bioeconomy.

## Abstract (precis)

Xylan is the major hemicellulosic polysaccharide in cereals and contributes to the recalcitrance of the plant cell wall toward degradation. *Bacteroidetes*, one of the main phyla in rumen and human gut microbiota, have been shown to encode polysaccharide utilization loci dedicated to the degradation of xylan. Here, we present the biochemical characterization of a xylanase encoded by a bacteroidetes strain isolated from the termite gut metagenome.

## INTRODUCTION

Xylan is the most abundant hemicellulose present in cell walls of higher plants, especially cereal grains and hardwoods (1). The xylan main chain is composed of β-1,4-linked d-xylopyranosyl (d-Xyl*p*) residues that can bear substitutions at O-2 and/or O-3 positions. l-arabinofuranosyl (l-Ara*f*), 4-*O*-methyl glucuronyl (d-MeGlcA*p*), and acetyl residues are frequent main-chain substituents, and l-Ara*f* moieties can be esterified by ferulate at their O-5 position. The nature of xylan backbone decorations varies depending on the species, the tissue, and the stage of development of the plant (2). Generally, graminaceous plants are rich in glucuronoarabinoxylan (GAX), while glucuronoxylan (GX) is found in dicots, the difference between these two categories being the relative amounts of l-Ara*f* and d-MeGlcA*p* present. Complete xylan degradation requires an extensive arsenal of enzymes that can act synergistically (3). The main chain is depolymerized by β-d-xylanases (EC 3.2.1.8) that hydrolyze internal β-1,4 bonds, while decorations are removed by a variety of accessory enzymes, including α-l-arabinofuranosidases (EC 3.2.1.55), α-d-glucuronidases (EC 3.2.1.139), feruloyl esterases (EC 3.1.1.73), and acetyl xylan esterases (EC 3.1.1.72). Finally, β-d-xylosidases (EC 3.2.1.37) break down xylooligosaccharides, removing d-Xyl*p* from the nonreducing end (4).

Xylanases are mainly found in the glycoside hydrolase (GH) families 5, 8, 10, 11, 30, and 43 in the CAZy database (www.cazy.org) (5). The GH10 family constitutes a monospecific family that includes only *endo*-xylanases. Enzymes from this family perform catalysis via a retaining mechanism (6), and their canonical three-dimensional (3D) structure is a TIM barrel, (β/α)_8_, which is the most commonly known (2,077 occurrences) protein fold in the Protein Data Bank (PDB) and which forms an active cleft able to accommodate up to seven xylosyl backbone units (7). In addition, according to the Pfam database (http://pfam.xfam.org/), 20 to 30% of β-d-xylanases are multidomain proteins, comprising catalytic domains associated with accessory or helper domains, such as carbohydrate binding modules (CBMs). The latter have been attributed various roles, including the ability to target specific regions in substrates (8), disrupt polysaccharide structure (9), or anchor enzymes to bacterial surfaces (10). In multidomain proteins, individual domains are defined as the structural, functional, or evolutionary units of proteins (11) and can be regarded as biological equivalents of components in complex devices whose parts can be interchanged. Mostly, domains in proteins are sequentially organized, with one domain following another one. However, around 10% to 20% of domain combinations are discontinuous, with one domain being inserted into another one (12).

Termites are wood-feeding animals that are considered an abundant source of biomass-degrading enzymes (13). Termites produce very few endogenous lignocellulose-degrading enzymes, and their gut microbiome is mainly responsible for their ability to capture nutrients and energy from plant biomass (14, 15). Over the last decade, numerous metagenomics studies revealed enzyme arsenals of termite gut microbiomes and detected promising enzymes for industrial use (16–20). Notably, Gram-negative *Bacteroidetes*, the dominant phylum in many animal digestive systems (21–25), utilize finely tuned glycan utilization systems. The paradigm for this type of system was provided by the well-studied starch utilization system (Sus) (26). In Sus-like systems, several proteins are encoded by genes found in a cluster (known as polysaccharide utilization loci, or PUL) and act in a coordinated manner to bind and hydrolyze complex sugars and utilize them for their metabolism (27). A xylan utilization system (Xus) that is composed of two outer membrane polysaccharide-binding proteins (XusB and XusD), two transporter proteins (XusA and XusC), and two outer membrane proteins (XusE and Xyn10C) was previously described in rumen and human digestive systems (28). Each of these proteins is expressed from a cluster of tandem genes that are organized as *xusA*-*xusB*-*xusC*-*xusD* (or sometimes only *xusC*-*xusD*), followed by *xusE* and *xyn10C*, the latter encoding a CBM-containing GH10 β-d-xylanase (28).

According to previous data, the CBMs in Xyn10C are inserted into the polypeptide sequence of the GH10 catalytic domain between structural elements β3 and α3 of the TIM barrel (29). The expression of Xyn10C was shown to be induced by xylan (30) along with the other xylanases, XynA and XynB. The most effective inducer is demonstrated to be a xylooligosaccharide with a degree of polymerization (DP) around 35, similar to the hydrolysates of Xyn10C (31). Altogether, this is consistent with the hypothesis that Xyn10C serves as a functional homologue of the Bacteroides thetaiotaomicron VPI-5482 SusG protein, initiating xylan metabolism through extracellular hydrolysis of polymeric substrates (28). In this regard, it has been proposed that Xyn10C is used as a functional marker of xylan degradation in the human gut (28, 30, 32). The potential roles and distributions of Xyn10C have recently attracted considerable attention but have not yet been fully described (29, 30, 32, 33).

**FIG 1:**
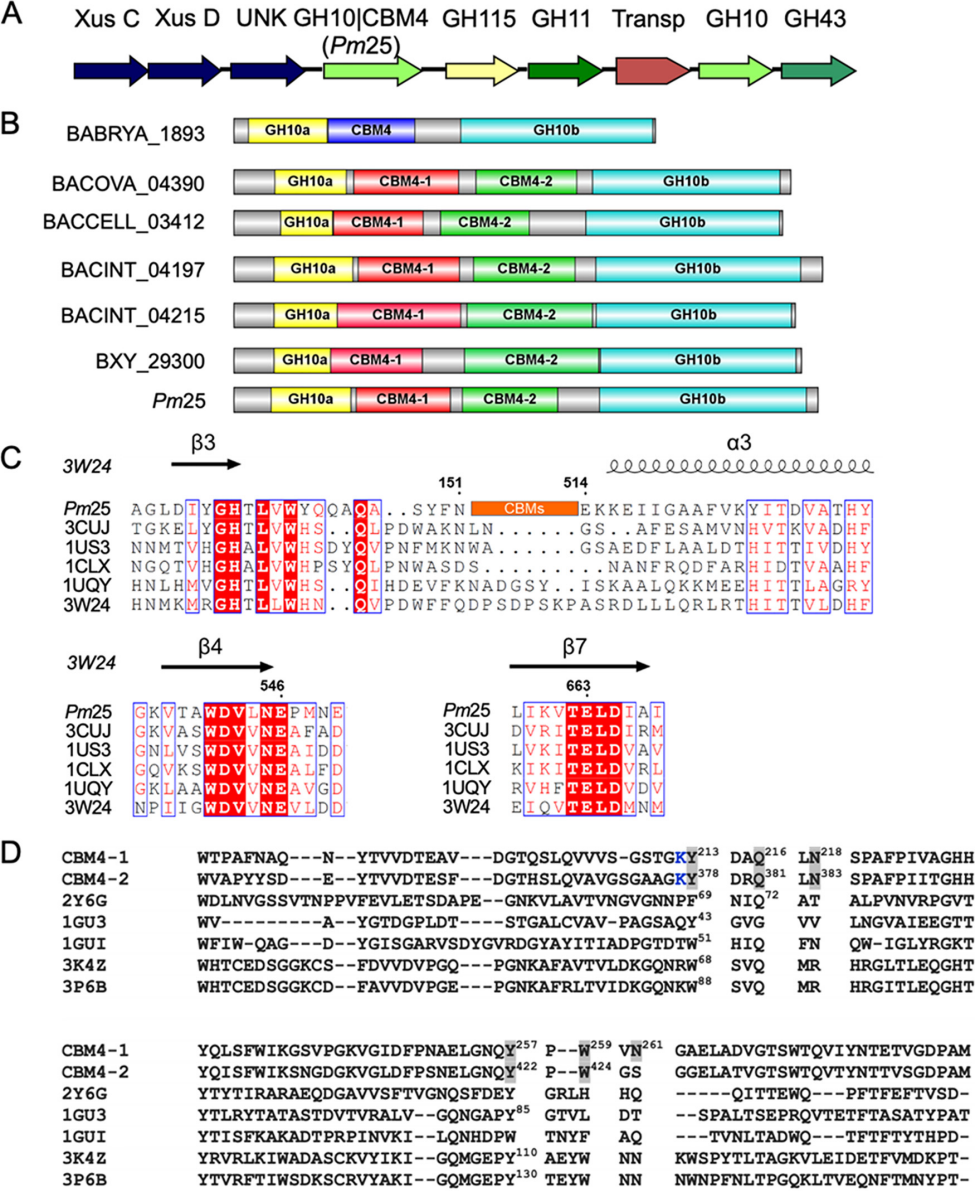
Modular architecture of Xyn10C-like protein. (A) Schematic representation of the putative xylan-active gene cluster. (B) Insertional architecture of Xyn10C-like proteins; the length of the amino acid is proportional to the length of the colored box. Shown are BABRYA_1893 (29), BACOVA_04390 (71), BACCELL_03412 (72), BACINT_04197 and BACINT_04215 (33), BXY_29300 (30), and *Pm*25 (17). (C) Amino acid alignment of *Pm*25 with other native GH10 xylanases, designated by their PDB codes: 3CUJ, Cellulomonas fimi xylanase/cellulase Cex (*Cf*Xyn10A); 1US3, Cellvibrio japonicus xylanase 10C; 1CLX, Pseudomonas fluorescens xylanase A; 1UQY, Cellvibrio mixtus xylanase B; 3W24, Thermoanaerobacterium saccharolyticum xylanase A. The β strands and α helices are shown above the aligned sequences. Numbering corresponds to the sequences of *Pm*25. Residues in red background are conserved, and those within blue frames are conservatively substituted. (D) Amino acid alignment of CBM4s in *Pm*25 with well-characterized CBM4s in the literature. CBM4-1 is the first CBM4 in *Pm*25; CBM4-2 is the second CBM4 in *Pm*25. PDB 2Y6G, CBM4 from Rhodothermus marinus xylanase; 1GU3, CBM4 from Cellulomonas fimi endoglucanase C; 1GUI, CBM4 from Thermotoga maritima laminarinase 16A; 3K4Z, CBM4 from Clostridium thermocellum cellulase CbhA; 3P6B, CBM4 from Clostridium thermocellum cellulase CelK. Residues highlighted in gray were used for mutagenesis. Numbered residues in PDB sequences indicate that they are responsible for ligand binding according to the literature. The K in blue is potentially labeled with *N*-hydroxysuccinimide dye, which was described in Materials and Methods for the MST experiment.

Previously, a putative *xus* locus assigned to the genus *Bacteroides*, Gram-negative anaerobic bacteria, was identified in a metagenomic library from the microbiome of a fungus-growing termite, *Pseudacanthotermes militaris* (17). This *xus* is composed of eight different open reading frames (ORFs) encoding putative XusC/D-like proteins, unknown protein (UNK), GH10 containing an insertion of two CBM4s (GH10|CBM4), GH115, GH11, a putative transporter protein, GH10, and GH43 (Fig. 1A). The GH10|CBM4 protein, designated *P. militaris* 25 (*Pm*25) here, presents an insertional modular structure homologous to Xyn10C protein (Fig. 1B). Here, we describe the characterization of *Pm*25 and discuss its activity with respect to its unusual multidomain organization. In addition, the potential function of the UNK was also investigated.

(This research was conducted by H. Wu in partial fulfillment of the requirements for a Ph.D. degree from Toulouse University [34].)

## RESULTS

### Bioinformatics analysis of the *Pm*25-encoding gene sequence.

Analysis of the primary amino acid sequence of *Pm*25 revealed a multimodular architecture composed of a signal peptide (residues 1 to 32), a GH10 catalytic domain (amino acid residues 66 to 151 and 514 to 753), and two putative tandem CBM4s (CBM4-1, residues 161 to 321, and CBM4-2, residues 324 to 486) that constitute insertion domains (Fig. 1B). Alignment of the amino acid sequence of *Pm*25 with those of other GH10 family members for which structural data are available revealed that CBM4-1 and CBM4-2 are inserted between strand β3 and helix α3 of the (β/α)_8_ structure (Fig. 1C). Moreover, this alignment allowed the identification of E546 and E663 as the putative catalytic acid/base and nucleophile, respectively.

Alignment of the amino acid sequences of CBM4-1/CBM4-2 with the well-characterized tandem CBMs, PDB entry 2Y6G from Rhodothermus marinus Xyn10A, 1GU3 from Cellulomonas fimi Cel9B, 1GUI from Thermotoga maritima Lam16, 3K4Z from Clostridium thermocellum Cbh9A, and 3P6B from Clostridium thermocellum Cel9K, revealed modest similarity (16/19,15/15, 16/19, 20/21, and 18/21% similarity, respectively). Moreover, using this similarity, we could assign possible ligand binding roles to residues Y213, Q216, and Y257 in CBM4-1 and Y378, Q381, and Y422 in CBM4-2 (35–40) (Fig. 1D). In addition, based on homolog modeling (unpublished data), W259 and W424 in CBM4-1 and CBM4-2, respectively, could be aligned to W102, located in the hydrophobic cleft of the Thermotoga maritima Lam16 CBM4, whose importance in binding was demonstrated by Boraston and colleagues (35).

### SSN analysis.

**FIG 2:**
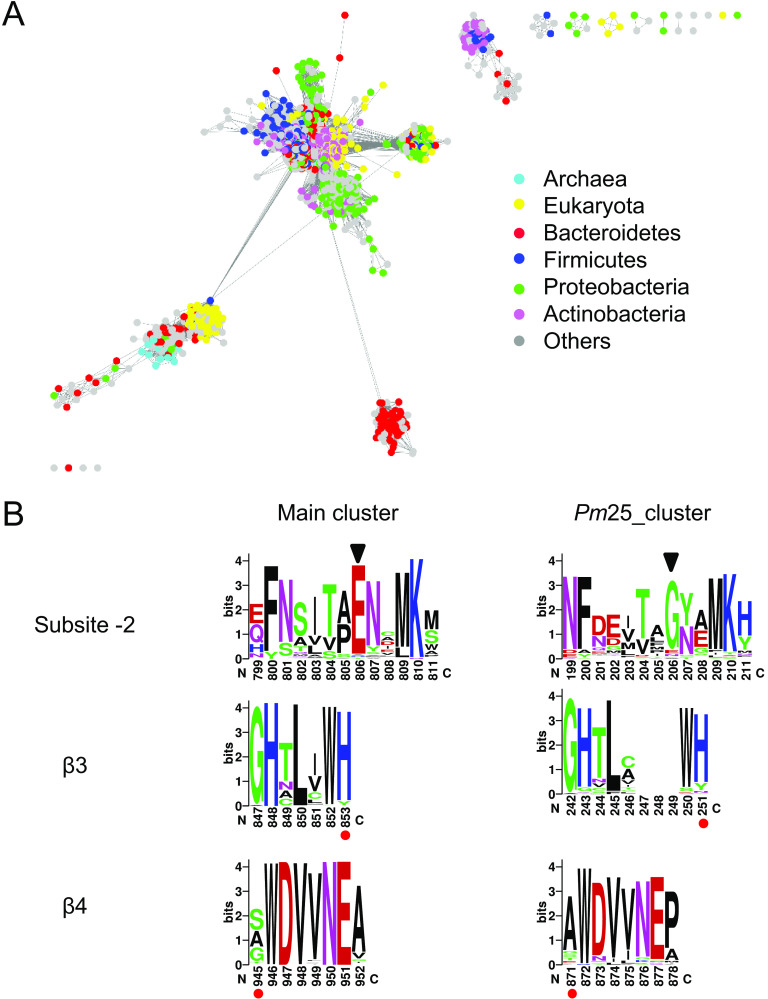
Sequence analysis of GH10 xylanase. (A) SSN of GH10 xylanase in CAZy database. The nodes are colored by the taxonomic annotation listed on the right. (B) Sequence logos of different motifs located at subsite −2, β3, and β4 of GH10 sequences. The length of amino acid sequence between β3 and β4 can be deduced from the sequence number with red dots underneath.

To identify *Pm*25 homologs and study their distribution, an SSN was created for all GH10 sequences (2,539 nonredundant sequences) present in the CAZy database (Fig. 2A). *Archaea*, *Eukaryota*, and *Bacteria* contribute 1, 16, and 81%, respectively, of GH10s, the remaining 3% being unassigned sequences. Bacterial GH10s belong mainly to four bacterial phyla, *Actinobacteria* (25%), *Bacteroidetes* (14%), *Proteobacteria* (14%), and *Firmicutes* (13%).

The SSN is centered on the main cluster, with other minor clusters scattered around. *Pm*25 is located in the bottom right cluster (*Pm*25_cluster), and all classified sequences (61 nodes) thereof are from *Bacteroidetes*.

To investigate the sequence differences between the *Pm*25_cluster and main cluster, sequences in each cluster were aligned before the sequence logo was constructed (Fig. 2B). The −2 subsite motif in *Pm*25_cluster is glycine instead of glutamate in the main cluster, and both glycine and glutamate at the −2 subsite were found in other enzymes (41–43). Importantly, the distance between the β3 motif and β4 motif is wider in *Pm*25_cluster (620 amino acids) than in the main cluster (92 amino acids), which indicated interrupted catalytic domains in *Pm*25_cluster.

To verify the relationship of *Pm*25_cluster with PUL, each sequence was searched against PULDB (http://www.cazy.org/PULDB/) (44). The majority of sequences (45 out of 61) were found in PUL. Among these, 44 out of 45 are always located downstream of hypothetical *susC*-*susD*-*unk* (see Table S2 in the supplemental material), suggesting that sequences in *Pm*25_cluster are Xyn10C-like proteins from the xylan utilization system in gut *Bacteroidetes*. The remaining sequences (16 out of 61) were not found in PUL, perhaps because of poor assignment.

### Enzyme optimal pH and temperature.

**FIG 3:**
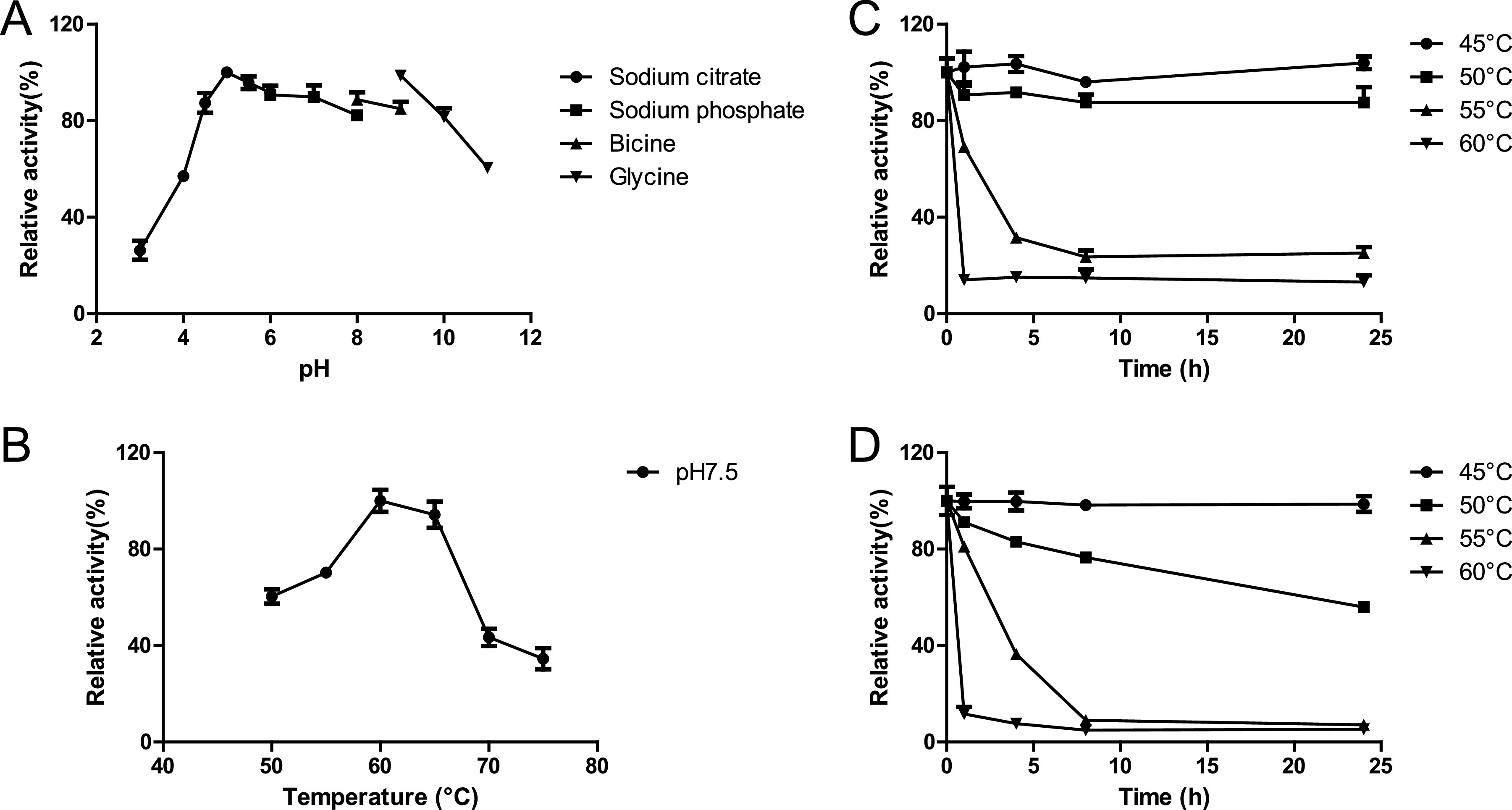
Optimal physiochemical parameters. (A) Optimum pH. (B) Thermoactivity at pH 7.5. (C) Thermostability at pH 7.5. (D) Thermostablity at pH 9.0.

The optimal pH of *Pm*25 was determined using beechwood GX as the substrate. The purified enzyme retained greater than 80% of its activity in the pH range from 4.5 to 9.0 (Fig. 3A). The activity was also measured at temperatures from 50 to 75°C, with maximum activity being observed at 60°C (Fig. 3B). However, since *Pm*25 was rather unstable at 60°C, thermostability was measured at both pH 7.5 and pH 9 (Fig. 3C and D, respectively). *Pm*25 remained fully active for over 24 h at either 50°C (pH 7.5) or 45°C (pH 9). Overall, optimal conditions for routine *Pm*25 assays were defined as pH 7.5 and 50°C.

### Enzyme assays and kinetic analysis.

**FIG 4:**
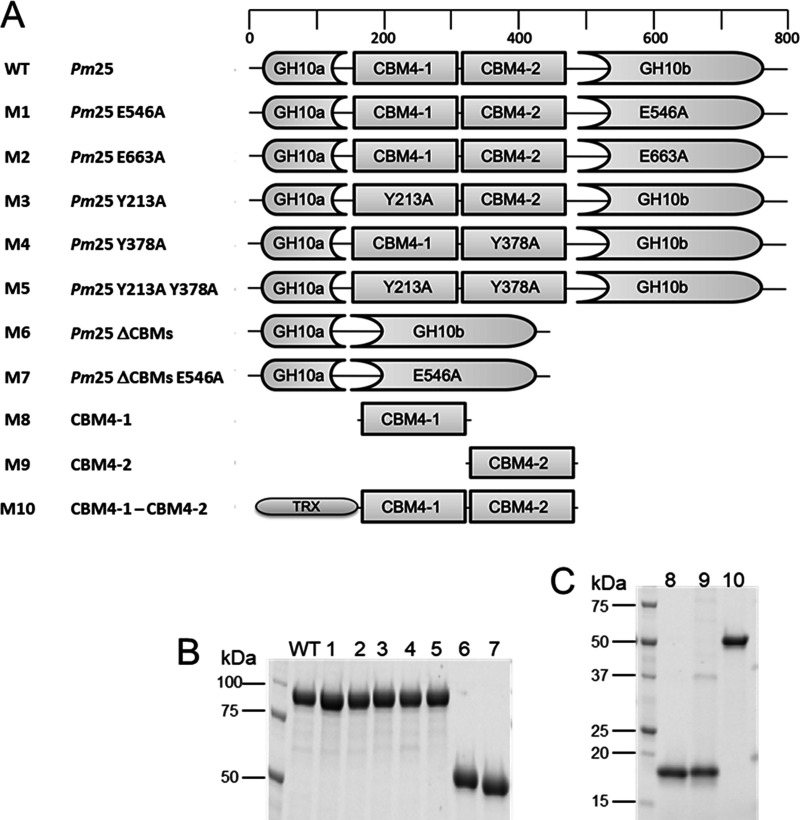
Mutational and truncation constructs. (A) Domain organization of *Pm*25 (WT) and modified constructs. (B) SDS-PAGE of purified WT and M1 to M7 constructs. (C) SDS-PAGE of purified CBM constructs M8 to M10.

Determining the hydrolytic activity of *Pm*25 on various substrates revealed that it is 3- to 4-fold more active on arabinoxylans (30 U·mg^−1^) than on beechwood GX (7.4 U·mg^−1^), indicating that the latter is the least suitable substrate among those tested (Table 1). When comparing the relative activities of *Pm*25 on different arabinoxylans, no significant differences were detected, although rye arabinoxylan (RAX), which possess more O-3 substitutions (45), qualifies as the best substrate tested. Testing the activity of the inactive mutants (M1 and M2) (Fig. 4) on different substrates revealed that they both displayed much lower (2 to 3 orders of magnitude) activity than *Pm*25 (Table S3).

**TABLE 1:** Kinetic parameters of *Pm*25

Substrate	Activity (U/mg)	*K_m_*_app_^*b*^ (mg/ml)	*K*_cat_ (s^−1^)	*K*_cat_/*K_m_* (min^−1^ mM^−1^)
Beechwood GX	7.4 ± 0.1	3.1 ± 0.2	10.3 ± 0.2	3.4 ± 0.1^*a*^
LVWAX	25.8 ± 0.8	2.0 ± 0.2	36.2 ± 1.1	18.0 ± 0.5^*a*^
ADWAX	21.7 ± 0.5	1.0 ± 0.1	30.4 ± 0.7	29.4 ± 0.7^*a*^
EDWAX	21.4 ± 0.4	1.7 ± 0.1	29.9 ± 0.5	18.1 ± 0.3^*a*^
RAX	30.3 ± 0.6	1.4 ± 0.1	42.4 ± 0.8	31.3 ± 0.6^*a*^
X_6_	—^*c*^	—	—	292.8 ± 36.8
X_5_	—	—	—	35.5 ± 2.4
X_4_	—	—	—	3.75 ± 0.1
*p*NPX_4_	—	—	—	2381.7 ± 381.7
*p*NPX_3_	—	—	—	1041.4 ± 138.2
*p*NPX_2_	—	—	—	14.09 ± 0.66

a For polysaccharide, *k*_cat_/*K*_*m* app_ is in s^−1^ mg·ml^−1^.

b *K_m_*_app_, apparent *K_m_*.

c —, Not determined due to inability to saturate the enzyme under experimental conditions.

### Hydrolysis product analysis with XOSs.

**FIG 5:**
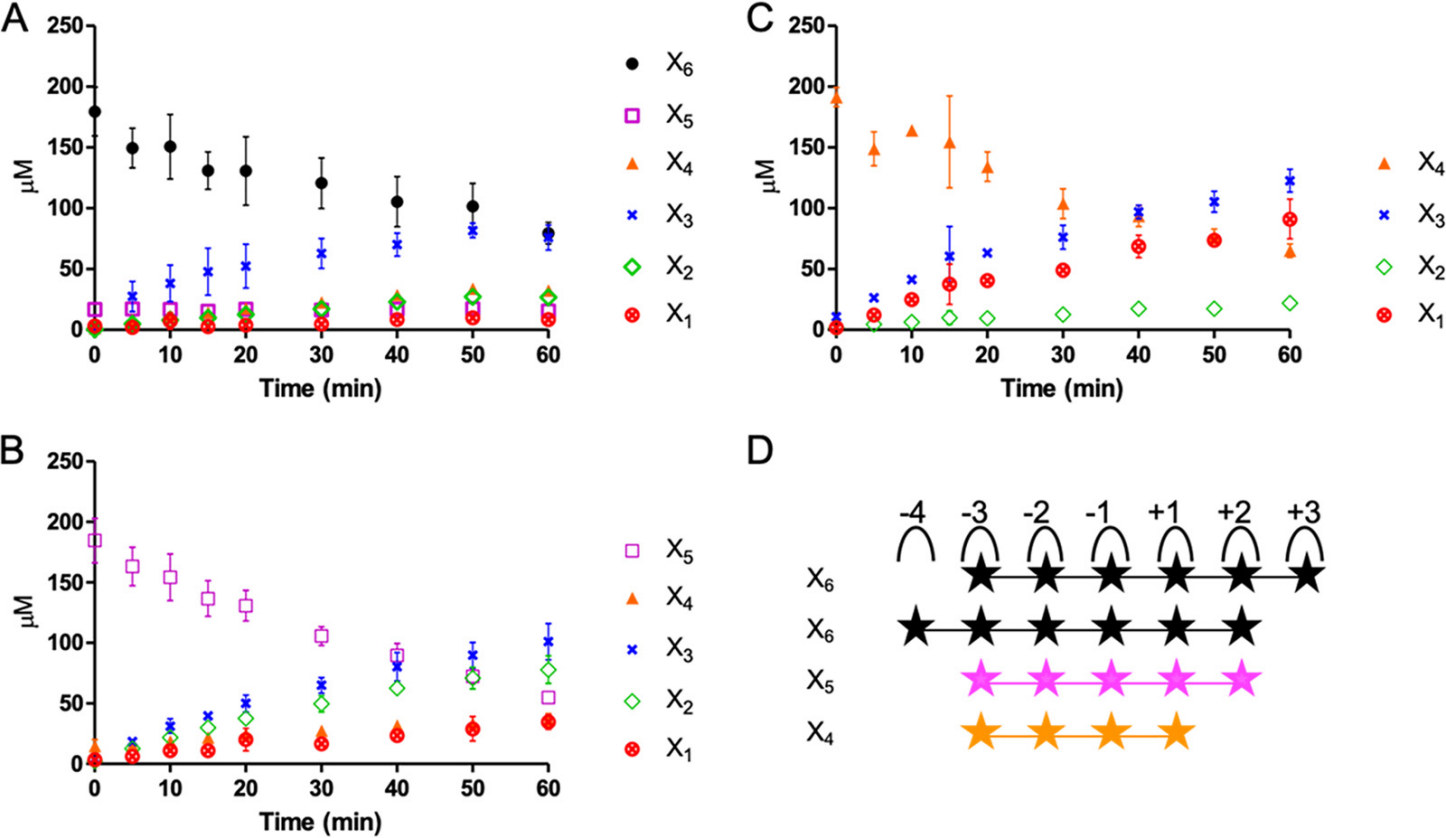
Progressive degradation of 0.2 mM XOS. (A to C) X6 (A), X5 (B), and X4 (C) by *Pm*25. (D) The potential subsite mapping of *Pm*25 with X6, X5, and X4.

HPAEC-PAD analysis of reaction mixtures containing different XOSs and *Pm*25 revealed that the hydrolysis of X_6_ (after 60 min) produced a mixture of X_2_, X_3_, and X_4_ at a ratio of 1:2:1, reaching 74 μM (Fig. 5A). Likewise, the hydrolysis of X_5_ led to the production of X_2_ and X_3_ as major products at 75 and 96 μM, respectively (Fig. 5B), while the hydrolysis of X_4_ yielded X_1_ and X_3_ (Fig. 5C) at 81 and 112 μM, respectively. Moreover, the activity of *Pm*25 was directly correlated with the DP of the XOS used, with higher-DP XOS leading to higher activity (Table 1). This suggests that the *Pm*25 substrate binding cleft is quite large and can accommodate at least six xylosyl residues. To further study binding cleft subsite interactions, reactions were performed using *Pm*25 and different aryl-β-xylosides. Accordingly, the catalytic efficiency (*k*_cat_/*K_m_*) obtained when using *p*-nitrophenyl-β-d-xylotetraose (*p*NPX_4_) as the substrate was approximately 2.3-fold greater than that measured when using *p*-nitrophenyl-β-d-xylotriose (*p*NPX_3_) (Table 1). These data provided the basis to calculate the binding affinity at the −4 subsite, revealing a value of 0.53 kcal/mol. Likewise, the −3 subsite binding affinity is 2.76 kcal/mol, which correlates with the fact that the *k*_cat_/*K_m_* value for the hydrolysis of *p*NPX_3_ is over 70-fold higher than that measured for the hydrolysis of *p*-nitrophenyl-β-d-xylobiose (*p*NPX_2_) (Table 1). Furthermore, the use of construct M6 (i.e., *Pm*25 devoid of CBMs) (Fig. 4) to hydrolyze aryl-β-xylosides revealed similar affinities at subsites −4 and −3 (0.75 and 2.69 kcal/mol, respectively), inferring that the two CBM4 domains do not influence catalysis when using these substrates.

### CBM and UNK ligand specificity.

To probe the function of CBM4-1 and -2, various constructions with CBM deletions or inactivations (M1 *Pm*25 E546A, M7 *Pm*25ΔCBMs E546A, M8 CBM4-1, M9 CBM4-2, and M10 CBM4-1-CBM4-2) were designed and expressed (Fig. 4), and affinity gel electrophoresis experiments were performed. Since the results of bioinformatics analysis putatively assign CBM4-1 and -2 to CBM family 4, initial tests were performed on xylan and glucan before performing complementary tests with other polysaccharides (Table 2). All proteins displayed strong affinity with the different xylans tested. However, very low or no affinity was detected for β-glucan and xyloglucan, and none of the proteins bound to arabinan, nanocellulose, and galactomannan (Table 2). Importantly, in the presence of different xylans, the dissociation constant (*K_d_*) of M10 (CBM4-1 and -2 in tandem) was much lower than that of M8 (CBM4-1 alone) and M9 (CBM4-2 alone), ranging from 3.2 to 9, 10.9 to 31.9, and 8.0 to 17.5 10^−2 ^mg·ml^−1^, respectively, suggesting that there is cooperativity between the two CBMs. Comparing the behavior of the inactive mutants M1 and M7 with that of M10 revealed that the affinity of the inactive *Pm*25 M1 for xylans was similar to that exhibited by M10 and the CBM4-1/-2 tandem, whereas the *K_d_* values obtained for the inactive enzyme devoid of CBMs (i.e., M7) were significantly higher. This suggests that xylan binding is largely driven by the two CBM4 domains. Comparing M8 and M9 revealed that M9 systematically exhibited lower (1.4 to 1.8 times) *K_d_* values for tests involving xylans, suggesting that its binding ability is stronger (Table 2). However, the results obtained with other polysaccharides indicate that CBM4-1 weakly binds to xyloglucan, while M9 (CBM4-2 alone) does not (Fig. S1). Overall, the results suggest that binding specificities of CBM4-1 and -2 are not identical.

**TABLE 2:** Binding of the recombinant proteins to soluble substrates as determined by affinity gel electrophoresis

Ligand	*K_d_* for^*a*^:				
	M1	M7	M8	M9	M10
RAX	2.8 ± 0.1	18.8 ± 3.5	31.9 ± 0.8	18.1 ± 2.2	5.1 ± 0.4
LVWAX	3.7 ± 0.3	27.1 ± 1.9	22.7 ± 1.1	15.9 ± 1.9	3.7 ± 0.2
ADWAX	2.3 ± 0.2	15.6 ± 0.2	10.9 ± 0.7	8.0 ± 0.1	6.4 ± 0
EDWAX	2.0 ± 0.1	16.7 ± 0.4	12.6 ± 0.8	9.2 ± 0.4	3.2 ± 0.0
Beechwood GX	4.9 ± 0.3	62.0 ± 1.9	30.3 ± 1.2	17.5 ± 0.1	9.0 ± 0.5
Nanocellulose	−	−	−	−	−
Galactomannan	−	−	−	−	−
Xyloglucan	+	+	+	−	+
Arabinan	−	−	−	−	−
β-Glucan	+	−	+	+	+

a The unit for *K_d_* is mg·ml^−1^; +, retardation observable; −, no retardation.

**FIG 6:**
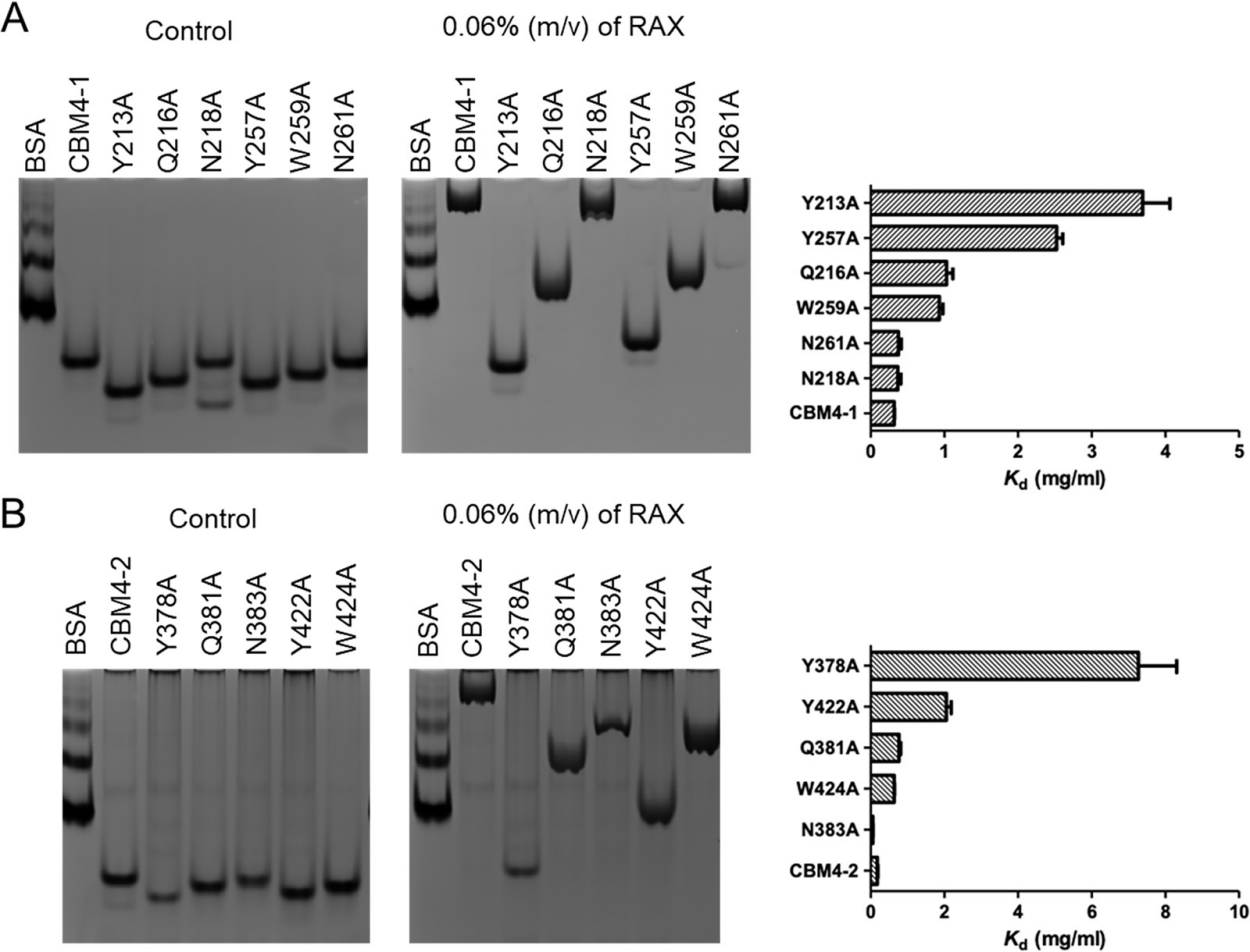
Affinity gel electrophoresis of different CBM mutations in *Pm*25. The control gel on the left lacks ligand, while the right one is with 0.06% (mol/vol) RAX. (A) Differential retardation of CBM4-1 (M8) and its mutants, CBM4-1 Y213A, Q216A, N218A, Y257A, W259A, and N261A. The *K_d_* values for each construct are on the right. (B) Differential retardation of CBM4-2 (M9) and its mutants, CBM4-2 Y378A, Q381A, N383A, Y422A, and W424A. The *K_d_* values for each construct are on the right.

To further probe the ligand binding ability of the two *Pm*25-associated CBM4 domains, different residues putatively involved in ligand binding were mutated (Fig. 6) based on sequence alignment (Fig. 1D) and structural comparison of CBM4 (unpublished data). Accordingly, the binding abilities of both CBM4-1|Y213A and CBM4-2|Y378A were lost, confirming that these residues play essential roles in the CBM-ligand interaction (Fig. 6). The binding abilities of CBM4-1|Y257A and CBM4-2|Y422A were also diminished, but ligand interactions were still observable, indicating that these tyrosines play less critical roles than Y213 and Y378, respectively (Fig. 6A and B). Likewise, other mutants, such as CBM4-1|Q216A, CBM4-1|W259A, CBM4-2|Q381A, and CBM4-2|W424A, also diminished, to some extent, ligand binding, but the mutation of asparagines (N218 and N261 in CBM4-1 and N383 in CBM4-2) had no apparent effect on binding, although these residues are close to the essential ones.

The determination of the *K_d_* values of M7, M8, and M9 for X_6_ was achieved using microscale thermophoresis (MST). The sigmoidal titration curves were used to calculate *K_d_* values (Fig. S2). The *K_d_* values obtained when using M7, M8, and M9 were 1.5 ± 0.2 mM, 4.7 ± 0.4 mM, and 1.8 ± 0.1 mM, respectively. Recalling that M7 is devoid of CBM domains, it is noteworthy that this construction displayed the highest binding ability, with M9 (i.e., CBM4-2) exhibiting a similar ligand binding ability.

Substrate depletion experiments performed using M1, M7, M8, and M9 on wheat bran revealed that the latter two constructions (CBM4-1 and CBM4-2, respectively) exhibited binding ability. Similarly, M1 (i.e., *Pm*25|E546A) was also able to bind to wheat bran despite its catalytic impotency (Fig. S3A). However, this was not the case for M7 (i.e., the inactivated catalytic domain alone), clearly demonstrating the role of the CBM in binding.

The affinity of UNK toward low-viscosity wheat arabinoxylan (LVWAX) was investigated. The retardation of the UNK band suggested the UNK can bind to xylan (Fig. S3B).

### Analysis of polysaccharide and wheat bran hydrolysis.

**FIG 7:**
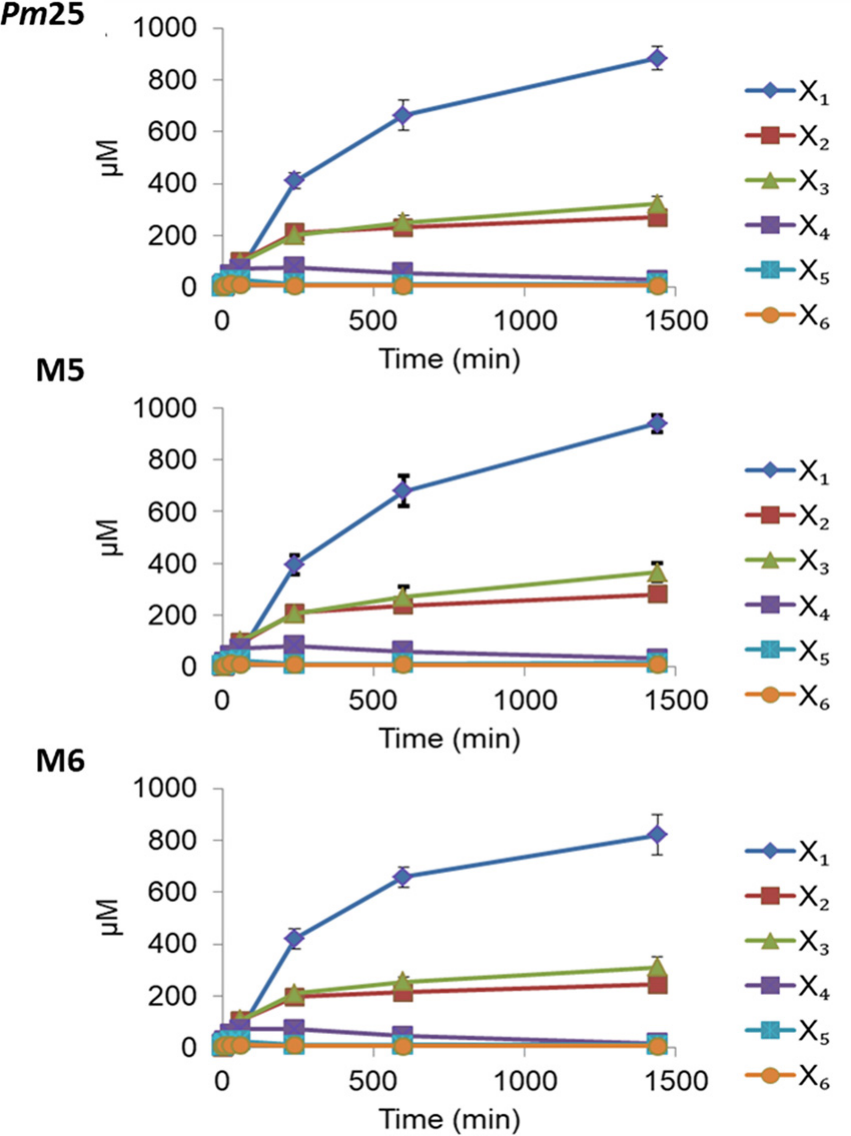
Hydrolysis of LVWAX by *Pm*25 and its mutants. The time-dependent degradation of LVWAX by *Pm*25 (WT), M5 (*Pm*25 Y213A Y378A), and M6 (*Pm*25ΔCBMs).

**FIG 8:**
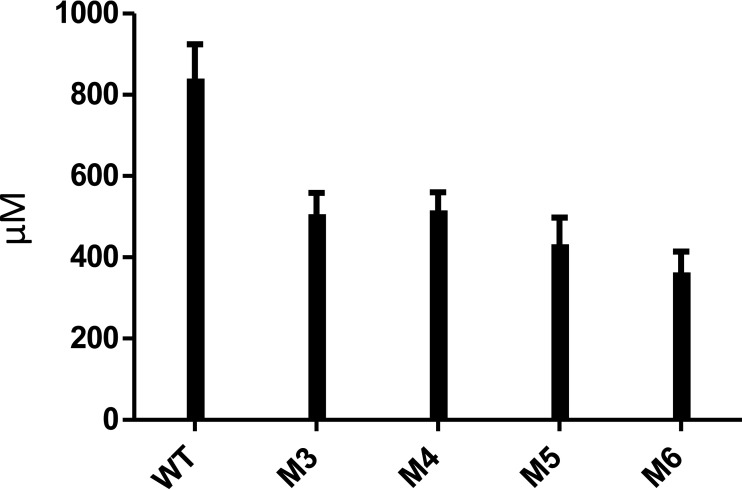
Hydrolytic patterns of *Pm*25 (WT) and mutants toward wheat bran. The release of xylooligosaccharides from wheat bran after overnight incubation with wild-type *Pm*25 compared to that of the mutants.

HPAEC-PAD of soluble polysaccharide hydrolysis mediated by *Pm*25 and its variants M5 and M6 failed to reveal significant differences in d-Xyl and XOS release (Fig. 7). This suggests that the CBM4 domains do not enhance degradation of soluble polysaccharides, since M6 is devoid of CBM4 domains. Performing a similar analysis using wheat bran as the substrate revealed that (after 14 h of incubation) d-Xyl and XOS release by *Pm*25 was approximately twice that of the variants M3, M4, M5, and M6 (Fig. 8). Significantly, in this experiment the consequences of the point mutations CBM4-1|Y213A and CBM4-1|Y378A were approximately equivalent to those produced by the ablation of the two CBM4 domains.

## DISCUSSION

Unlike the vast majority of multimodular enzymes that display a sequential arrangement of their modules, the enzyme described here is characterized by a discontinuous organization that involves the insertion of two CBM domains into one GH10 xylanase domain. In this regard, it is significant that the SSN analysis performed using the amino acid sequence of M6 replacing *Pm*25 located the sequence within the same cluster, even though the CBMs were omitted (data not shown). This suggests that the *Pm*25 GH10 domain forms part of a distinct group and implies that the intercalated GH10 arrangement is robust from an evolutionary standpoint. Moreover, the biochemical data described here demonstrate that, despite its discontinuous organization, *Pm*25 is a fully functional xylanase.

The first *Pm*25 analog was identified in a rumen-based member of the *Bacteroidetes* phylum (29). More have since been found in human gut bacteria (30, 32, 46), with *Pm*25 being the first described in termite gut. Several studies have revealed the importance of *Pm*25-like GH10 in xylan utilization systems (29, 30, 32, 33). Using SSN analysis, we have shown that *Pm*25-like xylanases are exclusively linked to *Bacteroidetes* and are mostly (44 out of 61 based on SSN analysis) adjacent to an *susC*-*susD*-(*unk*) cluster. This evidence of strong conservation is consistent with the fact that in their native host, the genes encoding *Pm*25 homologs are highly induced/expressed during growth on xylan (30, 46). In addition, our data show that the UNK protein upstream of *Pm*25 is a xylan-binding protein that strengthens the xylan utilization function of this core cluster (46), suggesting it is an analogue of SusE, which is also supported by the fact that like SusE, UNK is predicted to have a lipoprotein peptide signal by SignalP (47). Taken together, one can conclude that each component in the core cluster is essential for xylan utilization by members of the *Bacteroidetes* phylum in the gut ecosystem.

The *in vivo* function of *Pm*25 homologs in gut *Bacteroidetes* has not yet been fully established, although it has been suggested that it is a functional homolog of SusG (28). SusG is a cell surface-bound GH13 α-amylase that catalyzes the initial cleavage of polysaccharides (48). In our study, we also predict that *Pm*25 bears an N-terminal signal peptide that directs it to the cell surface, consistent with a proposal that was previously made for a *Pm*25 homolog (32). Moreover, SusG displays negligible activity compared to periplasmic α-amylases (48), an observation that is consistent with our findings. Indeed, compared to other xylanases (41, 49), both *Pm*25 and similar elements display quite poor catalytic efficiency toward polysaccharides (33, 50) and oligosaccharides (32). This trend is also observed in other polysaccharide-degrading systems, such as mannan utilization loci from members of the *Bacteroidetes* phylum (51) and the xylan-degrading system in the *Proteobacteria* (43) phylum. The underlying reason for such low activity most likely reflects its function. SusG-like proteins probably have a carbohydrate surveillance function, while highly active intracellular enzymes are charged with complete oligosaccharide breakdown prior to sugar catabolism. This clever and “selfish” strategy ensures that readily metabolizable sugars are not released into the environment, where they could be used by other bacteria that lack a specific glycan utilization machinery (52).

Remarkably, we found that *Pm*25 remains active over a broad pH range, maintaining more than 80% of its maximum activity at pH 9.0. This observation correlates well with results obtained for the *Pm*25 homologs Bacteroides intestinalis Xyn10C (*Bi*Xyn10C) and *Bi*Xyn10A, which were identified in the human gut microbiome (50). Accounting for the fact that alkaline-stable xylanases are sought after for use in applications such as paper pulp biobleaching, *Pm*25 might constitute a useful starting point for enzyme engineering aimed at improving its hydrolytic properties.

So far, we have been unable to obtain structural data pertaining to *Pm*25, and none is available for its closest homologs. Therefore, at this stage it is tricky to speculate on the exact topology and molecular determinants of its active site. Nevertheless, to gain some understanding, we have examined similarities with the family GH10 xylanase Cellvibrio japonicus Xyn10C (*Cj*Xyn10C), which displays approximately 30% identity to *Pm*25 and whose structure is known (PDB entry 1US3). Like *Pm*25, *Cj*Xyn10C exhibits rather poor activity on XOS, ascribed to weak substrate binding in subsite −2 (43). Unlike most other GH10 enzymes, *Cj*Xyn10C subsite −2 contains G295 in the place of E, whose side chain can hydrogen bond to the substrate. According to sequence alignment, *Pm*25 also lacks the vital E residue in subsite −2, an observation that might explain its poor ability to hydrolyze X_4_ (43, 53). Therefore, the −3 subsite with rather strong affinity value (2.76 kcal/mol) compared to others (53) is probably involved in the glycine subsite in the degradation of X_4_ to compensate for the poor −2 subsite. Taken together, a hypothetical subsite mapping of the active site of *Pm*25 with XOS is proposed for *Pm*25 (Fig. 5D).

The two CBM4s that are inserted into *Pm*25 clearly contribute to the binding and degradation of complex biomass. Our results reveal that this is especially true when both CBMs are functional and suggest that binding of large ligands involves a cooperativity phenomenon (Fig. 8). However, based on the PULDB database, the number of CBM domains in *Pm*25 homologs varies from one to three, and the CBMs are from different families, CBM4, CBM22, or unclassified. This suggests that the SusG-assimilated functions can be fulfilled by enzymes that are not configured in an identical way. Moreover, it also confirms that the TIM-barrel fold in the GH10 family is quite accommodating in terms of insertions at the β3/α3 loop.

Apparently, unlike many highly active periplasmic endoglucanases, such as SusA (48) and *Cj*Xyn10D (43), extracellular enzymes such as SusG, *Cj*Xyn10A, and *Cj*Xyn10C are generally appended to CBMs (43). Therefore, it is of interest to discuss the reason for this. CBM58 in SusG (54) and the CBM4s in *Pm*25 appear to improve the ability of the enzymes to hydrolyze insoluble substrates (Fig. 8), while CBM15 in *Cj*Xyn10C does not play an important role in catalysis, irrespective of whether the substrate is soluble or not (43). However, our data suggest that the affinity of *Pm*25 for soluble substrates was mostly derived from the binding ability of the CBMs (Table 2). In light of this observation, we propose that CBMs in membrane-associated enzymes temporarily withhold soluble oligosaccharides before their importation into the cell. This implies that the function of the CBM4 domains would be relatively independent of that of the GH10 domain. In this regard, it is noteworthy that the first structure of a SusG protein (54), which reveals that a CBM58 domain is inserted into the B domain of the GH13 α-amylase domain, reports that CBM58 does not form hydrogen bonds with the catalytic domain, an observation that argues in favor of an independent function. Regarding *Pm*25, evidence for an independent function of the CBM4 domains is provided by the fact that the xylan-degrading profile of the *Pm*25 wild type was almost identical to that of the CBM-deleted version, M6 (Fig. 7), and the fact that the xylan binding affinity of CBMs was relatively unaltered when the CBM domains were separated from the GH10 domain (Table 2). Finally, it is also useful to recall that the affinity values determined for subsites −4 and −3 of *Pm*25 and M6 were nearly identical. Therefore, we believe that the catalytic center of *Pm*25 and the binding surfaces of the CBM4 domains are disconnected, an organization that corresponds to independent functions and contributes to low enzyme reaction rates (55).

In conclusion, focusing on a termite gut-derived enzyme, we have provided further insight into the properties and function of Xyn10C-like enzymes that form part of core xylan utilization systems. This system seems to be rather efficient in terms of evolution, since it is conserved in termite gut, rumen, and human gut. Therefore, the role of the CBM insertion is an interesting question. In this respect, we have thoroughly succeeded in characterizing the enzyme and shown that the CBM4 domains can be successfully excised without loss of catalytic function. Regarding the enzyme’s substrate specificity, although it is difficult to speculate on the group of polysaccharides that might be preferential substrates in the termite gut environment, we have shown that it is better adapted for the hydrolysis of arabinoxylans than glucuronoxylans, which is consistent with the fact that the host termite feeds on crops such as sugarcane rather than wood.

## MATERIALS AND METHODS

### Materials.

Beechwood GX, d-Xyl, and most other reagents were purchased from Sigma-Aldrich (Darmstadt, Germany). Low-viscosity wheat arabinoxylan (Ara:Xyl = 38:62; LVWAX), acid-debranched wheat arabinoxylan (Ara:Xyl = 22:78; ADWAX), enzyme-debranched wheat arabinoxylan (Ara:Xyl = 30:70; EDWAX), rye arabinoxylan (Ara:Xyl = 38:62; RAX), galactomannan (carob; low viscosity), xyloglucan (tamarind), arabinan (sugar beet), β-glucan (barley; medium viscosity), xylobiose (X_2_), xylotriose (X_3_), xylotetraose (X_4_), xylopentaose (X_5_), xylohexaose (X_6_), *p*-nitrophenyl-β-d-xylopyranoside (*p*NPX), *p*-nitrophenyl-β-d-xylobiose (*p*NPX_2_), *p*-nitrophenyl-β-d-xylotriose (*p*NPX_3_), and *p*-nitrophenyl-β-d-xylotetraose (*p*NPX_4_) were all purchased from Megazyme (Bray, Ireland). Cellulose nanocrystals (nanocellulose) from cotton linters were prepared as previously described (56). Oligonucleotide primers were purchased from Eurogentec (Liège, Belgium) (Table 3).

**TABLE 3:** Primers used in this study

Target	Orientation^*a*^	Sequence (5′→3′)
Primers for site-directed mutagenesis		
*Pm25*|E546A	F	GTGGGATGTGCTGAACGCGCCCATGAACGAGAAC
	R	GTTCTCGTTCATGGGCGCGTTCAGCACATCCCAC
*Pm25*|E663tga	F	GGCAAACTCATCAAGGTGACGTGACTCGATATTGCCATATCCAC
	R	GTGGATATGGCAATATCGAGTCACGTCACCTTGATGAGTTTGCC
*Pm25*|tga663A	F	GCAAACTCATCAAGGTGACGGCGCTCGATATTGCCATATCC
	R	GGATATGGCAATATCGAGCGCCGTCACCTTGATGAGTTTGC
*Pm25*|H634A	F	GCATCGGCACGCAAATGGCGCTCAACCTCAACTGG
	R	CCAGTTGAGGTTGAGCGCCATTTGCGTGCCGATGC
CBM4-1|Y213A	F	GGCTCGACCGGAAAAGCGGACGCACAACTCAACTCTCC
	R	GGAGAGTTGAGTTGTGCGTCCGCTTTTCCGGTCGAGCC
CBM4-1|Q216A	F	CGGAAAATACGACGCAGCGCTCAACTCTCCGGCG
	R	CGCCGGAGAGTTGAGCGCTGCGTCGTATTTTCCG
CBM4-1|N218A	F	TACGACGCACAACTCGCGTCTCCGGCGTTCCCG
	R	CGGGAACGCCGGAGACGCGAGTTGTGCGTCGTA
CBM4-1|Y257A	F	CGGAACTGGGTAATCAAGCGCCATGGGTGAACGGC
	R	GCCGTTCACCCATGGCGCTTGATTACCCAGTTCCG
CBM4-1|W259A	F	GGTAATCAATATCCAGCCGTGAACGGCGCAGAACTGGC
	R	GCCAGTTCTGCGCCGTTCACGGCTGGATATTGATTACC
CBM4-1|N261A	F	CAATATCCATGGGTGGCGGGCGCAGAACTGGCCG
	R	CGGCCAGTTCTGCGCCCGCCACCCATGGATATTG
CBM4-2|Y378A	F	GGTGCGGCTGGCAAAGCGGACCGCCAACTCAAC
	R	GTTGAGTTGGCGGTCCGCTTTGCCAGCCGCACC
CBM4-2|Q381A	F	GGCAAATACGACCGCGCGCTCAACAGCCCGGCATTTCC
	R	GGAAATGCCGGGCTGTTGAGCGCGCGGTCGTATTTGCC
CBM4-2|N383A	F	GCAAATACGACCGCCAACTCGCGAGCCCGGCATTTCCG
	R	CGGAAATGCCGGGCTCGCGAGTTGGCGGTCGTATTTGC
CBM4-2|Y422A	F	GAACTCGGCAACCAGGCGCCGTGGGGAAGCGGC
	R	GCCGCTTCCCCACGGCGCCTGGTTGCCGAGTTC
CBM4-2|W424A	F	GGCAACCAGTATCCGGCCGGAAGCGGCGGCGAG
	R	CTCGCCGCCGCTTCCGGCCGGATACTGGTTGCC
Primers for in-fusion cloning		
*Pm25*_CBM4-1_L167-L320	F	CAGCCATATGGCTAGCTTGTGGACGGAAGTTTCGAAG
	R	GGTGGTGGTGCTCGATTAGAGGTCTGTGATCTCGACG
*Pm25*_CBM4-2_D332-D484	F	CAGCCATATGGCTAGCCTTGTGGACGGCAATTTCGAAGGCGGAG
	R	GGTGGTGGTGCTCGAGTTATTAGAGGTCGGTGACCTCAACGGCGTC
*Pm25*_CBM4-1 + 2_L167-D484	F	GACGACGACGACAAGCTTGTGGACGGAAGTTTCGAAGAGGGGATG
	R	GTGGTGGTGCTCGAGGTCGGTGACCTCAACGGCGTCGATC
pET32a_linearization	F	CTCGAGCACCACCACCACCACCAC
	R	CTTGTCGTCGTCGTCGCCAGAACCAGAACCGGCCAGGTTAG

a F, forward; R, reverse.

The GenBank accession number for the clone containing *Pm*25 is HF548280.1, and the protein ID for *Pm*25 is CCO21036.1.

### Bioinformatics analysis.

Putative signal peptide sequence analysis was performed using the SignalP 4.1 server (47). The domain annotation of *Pm*25 was done using InterPro protein sequence analysis (https://www.ebi.ac.uk/interpro/) with accession number S0DFK9. Multiple-protein sequence alignment of CBM4s was done using Clustal Omega (https://www.ebi.ac.uk/Tools/msa/clustalo/), and the alignment of the secondary structure elements of *Pm*25 with other structurally characterized GH10 family members was achieved using both Clustal Omega and ESPript 3 at http://espript.ibcp.fr/ESPript/ESPript/ (57).

### SSNs.

Amino acid sequences of GH10 family members were extracted from the CAZy database (http://www.cazy.org/GH10_all.html), updated on 29 May 2020. To remove redundant sequences, the 4,936 sequences were winnowed down to 2,539 by a sequence identity cutoff of 0.9 (58), length cutoff of 250, and fragment exclusion (59). Sequence similarity networks (SSNs) were constructed using the Enzyme Function Initiative Enzyme Similarity Tool (EFI-EST) (59) and visualized using Cytoscape 3.6 (60). The alignment score threshold was set to 35% sequence similarity, since nodes are linked with the edge when they share over 35% identity and each node represents one protein sequence. Multiple-sequence alignment of GH10s in different clusters was done by MAFFT (https://www.ebi.ac.uk/Tools/msa/mafft/), and sequence logos were constructed via WebLoGo (https://weblogo.berkeley.edu/logo.cgi).

### Cloning and site-directed mutagenesis.

Cloning of the plasmid pDEST17 containing *Pm*25 was achieved as described previously (61). All mutants were constructed using the QuikChange site-directed mutagenesis kit (Strategene, La Jolla, CA, USA) with oligonucleotide primers (Table 3). The M6 construct, which corresponds to *Pm*25 deprived of its CBMs, was obtained by gene synthesis (NZYTech, Lda, Portugal). The mutation of E546 to A in M6 yielded M7, while M8 and M9 were constructed by cloning the sequences encoding CBM4-1 and CBM4-2, respectively, into pET28a(+) expression vector. Likewise, the construct M10 is the pET32a(+) expression vector containing the sequence encoding both CBM4-1 and CBM4-2 cloned in frame with the thioredoxin tag using the In-Fusion cloning kit (Clontech, TaKaRa, Shiga, Japan). The DNA sequence of UNK (UniProt ID S0DDM9) deprived of its signal peptide sequence, as identified by the SignalP 4.1 server (47), was synthesized and subcloned into pET28a between the NheI and XhoI restriction sites.

### Protein expression and purification.

Wild-type *Pm*25 and the mutants M1, M2, M3, M4, and M5 (Fig. 4A) were expressed in Escherichia coli Rosetta(DE3) pLysS grown in ZYP autoinduction medium (62) at 25°C overnight. Constructs M6 to M10 and UNK were transformed into E. coli Tuner(DE3) and cultured for 2 h at 37°C until the optical density at 600 nm (OD_600_) reached 0.6. At this point, isopropyl-β-d-thiogalactopyranoside (IPTG; 200 μM final concentration) was added and growth was pursued at 16°C overnight. Cell pellets were collected by centrifugation, washed, and lysed using sonication (Fisherbrand Q700; tip diameter, 13 mm; output, 40 W), and the clarified cell lysates were applied to TALON metal affinity resin (Clontech, Mountain View, CA, USA). After elution, protein purity was estimated by SDS-PAGE to be 95%. Protein concentrations were determined by measuring absorbance at 280 nm and applying the Beer-Lambert equation. Theoretical molar extinction coefficients were calculated using ProtParam online software (63).

### Determination of pH and temperature optima.

The apparent optimal pH of *Pm*25 was determined in the pH range of 3.0 to 11.0, measuring the enzyme activity (0.4 μM final enzyme concentration) on 1% (mol/vol) beechwood GX at 37°C. The buffers used were 50 mM citrate buffer for pH 3.0 to 6.0, 50 mM phosphate buffer for pH 6.0 to 8.0, 20 mM bicine buffer for pH 8.0 to 9.0, and 20 mM glycine-NaOH for pH 9.0 to 11.0. Xylanase activity was determined by measuring the release of reducing sugars using the 3,5-dinitrosalicylic acid (DNS) assay (64, 65). Reactions were performed in triplicate at 37°C in the different buffers from pH 3.0 to 11.0, containing bovine serum albumin (BSA; 1 mg/ml). At regular intervals (0, 3, 6, 9, 12, 15, 18, 21, and 24 min), 100 μl of the reaction mixture was removed and added to 100 μl of DNS and kept on ice until all samples were ready. All samples then were heated at 95°C for 10 min and cooled on ice before adding 1 ml of deionized water and recording the absorbance at 540 nm using a spectrophotometer. A d-xylose series (0 to 1 mg/ml) was used to prepare a standard curve. The apparent optimal temperature was determined over the range of 21 to 90°C in 50 mM phosphate buffer (pH 7.5). Thermostability was monitored by preincubating the enzyme in the absence of substrate in 50 mM phosphate buffer (pH 7.5) at 45, 50, 55, and 60°C from 0 to 24 h. Residual enzyme activity in each case was then assayed as described above.

### Enzyme specificity and kinetics.

Enzyme kinetics were measured using a *Pm*25 concentration of 0.4 μM and 0.08 μM to degrade beechwood GX and other soluble polysaccharides, respectively. Initial rates (the concentration of d-Xyl equivalent released, in milligrams per milliliter per minute) were determined using a range of substrate concentrations (from 0.5 to 40 g/liter beechwood GX and 0.25 to 10 g/liter for other soluble substrates) under optimal conditions. The DNS assay was used to monitor reducing sugar release as described earlier. The kinetic parameters (*k*_cat_ and apparent *K_m_*) were calculated using nonlinear regression in SigmaPlot 11.0 (Systat Software, San Jose, CA, USA). One unit of xylanase activity was defined as the amount of enzyme that catalyzes the release of 1 μmol of d-Xyl equivalents per min.

To study the hydrolysis of xylooligosaccharides (XOS) with a degree of polymerization of 4 to 6 (X_4_ to X_6_), reactions were performed using various concentrations (0.05 to 0.8 mM) and the optimal reaction conditions. Assays began upon the addition of enzyme, its final concentration being fixed to account for the nature of the substrate. Accordingly, 2.60, 0.26, and 0.026 μM enzyme were used for X_4_, X_5_, and X_6_, respectively. At regular intervals (0, 5, 10, 15, 20, 30, 40, 50, and 60 min), aliquots were removed and immediately heated at 95°C for 10 min to stop the reaction. The hydrolyzed products were then analyzed by high-performance anion-exchange chromatography with pulsed amperometric detection (HPAEC-PAD) using an ICS 3000 dual device (Dionex, France) equipped with Carbo-Pac PA-100 guard and analytical columns (2 by 50 mm and 2 by 250 mm, respectively) as described before (65). Ten microliters of sample was injected, and separation was achieved by applying a gradient of 0 to 85 mM sodium acetate, 150 mM NaOH from 0 to 30 min, isocratic elution with 500 mM sodium acetate, 150 mM NaOH from 30 to 33 min, and reequilibration of the column with 50 mM sodium acetate, 150 mM NaOH for another 10 min at a flow rate of 0.25 ml/min. Calibration was achieved using d-Xyl and XOS (X_2_, X_3_, X_4_, X_5_, and X_6_) at concentrations from 5 to 50 μM. Plotting the hydrolysis rate (micromolars per minute) versus oligosaccharide substrate concentration (micromolars) yielded a linear relationship, meaning that the catalytic constant *k*_cat_/*K_m_* could be calculated from the slope *k* using equation 1, where [*E*] is the final concentration of enzyme.

(1) $$k=k_{\text{cat}}/K_{m\;\times }\left[E\right]$$

All experiments were performed in triplicate, and reported values are the means from three experiments.

### Determination of subsite affinities.

The binding affinities of glycone subsites were calculated using equation 2 (66, 67).

(2) $$A_{-i}=\frac{\mathrm{RT}}{4,183}\ln\frac{{\left(\frac{k_{\text{cat}}}{K_{m}}\right)}_{\left(\mathrm{pNP}X_{i}\right)}}{{\left(\frac{k_{\text{cat}}}{K_{m}}\right)}_{\left(\mathrm{pNP}X_{i-1}\right)}}({\text{kcal×mol}}^{-1})$$

where *A_−i_* is the subsite affinity at *−i* subsite, *k*_cat_/*K_m_* of *p*NPX*_i_* is the performance constant for *p*NP-labeled XOS with a DP of *i* (where *i* is a whole number), and *R* is the universal gas constant (8.314 J mol^−1^ K^−1^).

To determine the catalytic parameters of reaction mixtures containing pNP-XOS, the final concentration of *Pm*25 used was 6, 27, and 136 nM for *p*NPX_4_, *p*NPX_3_, and *p*NPX_2_, respectively. Similarly, the final concentration of M6 was 10 nM for *p*NPX_4_ and *p*NPX_3_ and 54 nM for *p*NPX_2_. The concentration range of substrate was 0.025, 0.05, and 0.1 mM for *p*NPX_3_ and *p*NPX_4_ and 0.5, 2, and 5 mM for *p*NPX_2_. All experiments were performed in duplicate. The plot of hydrolysis rate against *p*NP substrate concentration was linear, which indicated that substrate concentration was far below the *K_m_*. Therefore, the *k*_cat_/*K_m_* of reaction mixtures containing aryl β-xylosides was determined under optimum conditions using equation 3 (53, 66). Briefly, the substrate concentrations at the beginning of the reaction ([*S*_0_]) and at specific times ([*S_t_*]) were fitted to equation 3, where *k* = (*k*_cat_/*K_m_*) [Enzyme] and [Enzyme] is the final concentration of enzyme.

(3) $$k=\ln\left[S_{0}\right]/\left[S_{t}\right]$$

The molar extinction coefficient of *p*NP (15,570 M^−1^·cm^−1^) was determined experimentally by measuring the absorbance at 404 nm for a standard curve ranging from 0 to 0.12 mM *p*NP at pH 7.5 and 50°C.

### AGE.

The binding of CBM4-1 and CBM4-2 to soluble polysaccharides was evaluated by affinity gel electrophoresis (AGE), using 7.5% (mol/vol) acrylamide gels containing various amounts of polysaccharide (for the concentration range of RAX, refer to Table S1 in the supplemental material). ADWAX, EDWAX, and LVWAX samples and beechwood GX were used at 0.006 to 0.06% (mol/vol), while other polysaccharides were used at 0.5% (mol/vol). Pure protein (6 μg) was migrated (10 mA/gel for about 1 h at room temperature) on gels in 25 mM Tris, 250 mM glycine buffer, pH 8.3. BSA (15 μg) was also included in the experiment as a negative, noninteracting control. Proteins were visualized by Coomassie blue staining. The dissociation constant *K_d_* was calculated as previously described (68). In equation 4,

(4) $$\frac{1}{R_{0}\;-\;r}=\frac{1}{R_{0}\;-\;R_{c}}(1\;+\;\frac{K}{c})$$

*R*_0_ is the relative protein migration distance compared to that of BSA in the control gel (without ligand). The variable *r* is the relative protein migration distance compared to that of BSA in ligand-containing gels. *R_c_* is the relative protein migration distance of complex between protein and ligand. *c* is the concentration of ligand. When equation 4 was plotted, taking 1/(*R*_0_ − *r*) as the ordinate and 1/*c* as the abscissa, a straight line was obtained. The intercept of the line on the abscissa provided a negative reciprocal value of the dissociation constant (−1/*K*). All experiments were performed in triplicate, and reported values are the means from three experiments.

### MST.

Microscale thermophoresis (MST) (69) was carried out on a Monolith NT115 (NanoTemper Technologies GmbH, Munich, Germany) at 25°C, 20% light-emitting diode (LED) power, and 40% MST power. Protein samples were labeled at a final concentration of 10 μM, as previously described (68). An aliquot of 0.6 μM labeled protein was mixed with decreasing concentrations of cellulose nanocrystals (from 312.5 mg·liter^−1^ to 0.3 mg·liter^−1^) in either buffer 1 (50 mM Tris-HCl, pH 7.4, 150 mM NaCl, 10 mM MgCl_2_, 0.05% Tween 20) or buffer 2 (50 mM sodium phosphate buffer, pH 7, and 0.05% pluronic acid). Data analysis was performed with MO Affinity software (NanoTemper). The Hill equation was chosen to determine a value for the 50% effective concentration (EC_50_).

To fluorescently label the amine groups of exposed lysines (Fig. 1D) lying in the vicinity of the ligand binding clefts in M7, M8, and M9, 100 μl of pure proteins (20 μM) was treated with the reagents in the protein labeling kit (RED-NHS) by following the manufacturer’s instructions. The labeled proteins were recovered and purified using TALON metal affinity resin, and the concentration of the labeled proteins was estimated using SDS-PAGE and serial dilutions of a protein solution of known concentration. A total of 16 dilutions (350 to 11 mM) of a solution of X_6_ containing either 0.075 μM M7, 0.13 μM M8, or 0.07 μM M9 in 50 mM phosphate buffer, pH 7, 0.05% Pluronic F-127 were loaded onto 16 standard capillaries. The initial fluorescence of all 16 samples was obtained by performing a capillary scan with LED power of 25% for M8 and 20% for M7 and M9. The dissociation constant *K_d_* was calculated by selecting the tab “Initial Fluorescence Analysis Set” in the Affinity Analysis software (70). To perform the SDS denaturation (SD) test, 10 μl of samples 1 to 3 and 14 to 16 were mixed with 10 μl of 4% SDS, 40 mM dithiothreitol (DTT) after 10 min centrifugation at 15,000 × *g*, followed by a 5-min incubation of the mixture at 95°C to denature the protein. The samples then were loaded into the capillaries to measure their fluorescence intensities.

### Solid depletion assay.

The ability of inactivated M1, M7, M8, and M9 to bind wheat bran was investigated by incubating 100 μg of protein with 4 mg of wheat bran in 200 μl of reaction buffer (50 mM sodium phosphate, pH 7). Reactions were performed in 0.2-ml PCR tubes and incubated at 10°C for 2 h with agitation in an Eppendorf Thermomixer R at 1,400 rpm. For each reaction, the supernatant containing the unbound enzyme fraction was recovered after centrifugation using a benchtop microcentrifuge. The pelleted substrate was washed 3 times with reaction buffer. Finally, 20 μl of Laemmli sample buffer was added to the pellet and heated at 95°C for 10 min to denature the protein (bound fraction). All the fractions were verified by SDS-PAGE. BSA was used as a negative control.

### Hydrolysis of wheat arabinoxylan and wheat bran.

Product profiles were generated with *Pm*25, M3, M4, M5, and M6 on either LVWAX (0.5% [mol/vol]) or wheat bran (20 mg/ml of wheat bran prehydrated for 12 h at 37°C, 1,400 rpm using the Eppendorf Thermomixer R). The enzymes (final concentration, 0.5 μM) were incubated with the respective substrate in 50 mM phosphate buffer (pH 7.5) and 1 mg/ml BSA. Enzymatic reaction mixtures were incubated at 37°C for either 24 h for LVWAX or 14 h for wheat bran, and aliquots were removed at regular time intervals and heated at 95°C for 10 min to terminate the reaction. Each sample was centrifuged at 20,000 × *g* for 5 min and quantified by HPAEC-PAD on a Dionex PA1 column equipped with a Carbo-Pac PA-1 guard and analytical columns (4 by 50 mm and 4 by 250 mm, respectively). Separation of oligosaccharides was achieved by isocratic elution with 100 mM NaOH at a flow rate of 1 ml/min from 0 to 5 min, a gradient of 0 to 120 mM sodium acetate in 100 mM NaOH from 5 min to 25 min, and isocratic elution with 500 mM sodium acetate in 100 mM NaOH from 25 min to 35 min. The column then was reequilibrated with 100 mM NaOH for another 10 min. Calibration was achieved using d-Xyl and XOS (X_2_, X_3_, X_4_, X_5_, and X_6_) at concentrations from 5 to 100 μM.

## Supplementary Material

Supplemental file 1
