# Supplementary material for: Multimodularity of a GH10 Xylanase Found in the Termite Gut Metagenome
Source: Appl Environ Microbiol. 2021 Jan 15;87(3):e01714-20. doi: 10.1128/AEM.01714-20 (PMC7848910; doi:10.1128/AEM.01714-20)
Supplement: Supplemental file 1 [file AEM.01714-20-s0001.pdf]

SUPPLEMENTARY MATERIAL:

Materials and Methods:

Enzyme specificity of *Pm*25 and mutants thereof. To compare the specific activity of *Pm*25 and mutants thereof, RAX (0.75%, m/v), LVWAX (0.4%, m/v) and beechwood GX (1%, m/v) were employed. The final concentration of *Pm*25 used to degrade beechwood GX and other soluble substrates is 35 and 7 mg/L, respectively. Owing to the weaker activity of the mutants (M1 and 2), for activity assays the final enzyme concentration was increased 100-fold compared to assays using the wild type *Pm*25.

Table S1. RAX concentration series used for different constructs.

RAX‰ (m/v)	#1	#2	#3	#4	#5	#6	#7	#8	#9	#10	#11	#12
M8	0.9	0.6	0.3	0.15	0.105	0.06	0.03					
M8_Y213A	2.1	1.5	1.2	0.9	0.6	0.3	0.15	0.105	0.06	0.03		
M8_Q216A	1.5	1.2	0.9	0.6	0.45	0.3	0.15	0.105	0.06	0.03		
M8_N218A	0.9	0.6	0.3	0.15	0.105	0.06	0.03					
M8_Y257A	2.1	1.5	1.2	0.9	0.6	0.3	0.15	0.105	0.06	0.03		
M8_W259A	1.5	1.2	0.9	0.6	0.3	0.15	0.105	0.06	0.03			
M8_N261A	0.9	0.6	0.3	0.15	0.105	0.06	0.03					
M9	1.5	1.2	0.9	0.6	0.3	0.15	0.126	0.105	0.06	0.03		
M9_Y378A	3	2.1	1.5	1.2	0.9	0.6	0.45	0.3	0.15	0.105	0.06	
M9_Q381A	1.5	1.2	0.9	0.6	0.3	0.15	0.105	0.06				
M9_N383A	1.5	1.2	0.9	0.6	0.3	0.15	0.126	0.105	0.09	0.06		
M9_Y422A	2.1	1.5	1.2	0.9	0.6	0.45	0.3	0.15	0.105	0.06		
M9_W424A	1.5	1.2	0.9	0.6	0.3	0.15	0.105	0.06				
M11	0.45	0.3	0.15	0.126	0.105	0.09	0.06	0.045	0.03	0.0225	0.015	0.01
M1	0.45	0.3	0.15	0.126	0.105	0.09	0.06	0.045	0.03	0.0225	0.015	0.01
M8	0.45	0.3	0.15	0.126	0.105	0.0225	0.015					

Table S2. Relationship of GH10 sequences in *Pm*25_cluster and the PULDB.

No.	Uniprot ID or Genbank ID	Protein Name	Organism name/NCBI id	PUL number	PULDB link
1	A0A5C0VIA8	FYC62_09295	Pedobacter sp. CJ43 - †2605747	CAZyme cluster†5	http://www.cazy.org/PULDB/index.php?prot=FYC62_09295
2	A0A0P0GGQ1	BcellWH2_04323	Bacteroides cellulosilyticus WH2 (new assembly) - 1268240	Literature-derived PUL 104	http://www.cazy.org/PULDB/index.php?prot=BcellWH2_04323
3	ALJ47172.1	BACOVA_00247(Bovatus_02547)	Bacteroides ovatus ATCC 8483 - 411476	Literature-derived PUL 4	http://www.cazy.org/PULDB/index.php?prot=BACOVA_00247
4	A0A0P0GM57	AA416_02680(BcellWH2_00959)	Bacteroides cellulosilyticus WH2 (new assembly) - 1268240	Literature-derived PUL 6	http://www.cazy.org/PULDB/index.php?prot=BcellWH2_00959
5	ALJ48332.1	BACOVA_03431(Bovatus_03727)	Bacteroides ovatus ATCC 8483 - 411476	Literature-derived PUL 73	http://www.cazy.org/PULDB/index.php?prot=BACOVA_03431
6	A0A3D2M3F9	DHW41_19925(Bovatus_01728)	Bacteroides ovatus ATCC 8483 (new assembly) - 411476	Literature-derived PUL 93	http://www.cazy.org/PULDB/index.php?prot=Bovatus_01728
7	A0A4P7VLL0	E7746_01620	Muribaculum sp. TLL-A4 - 2530390	Predicted PUL 1	http://www.cazy.org/PULDB/index.php?prot=E7746_01620
8	A0A4P8RNL4	C1N53_08480	Pontibacter sp. SGAir0037 - 2571030	Predicted PUL 13	http://www.cazy.org/PULDB/index.php?prot=C1N53_08480
9	A0A1L6R297	AO058_06920	Salegentibacter sp. T436 - 1729720	Predicted PUL 14	http://www.cazy.org/PULDB/index.php?prot=AO058_06920
10	A0A4P7W8B6	E7747_14485	Muribaculum sp. H5 - 2530393	Predicted PUL 17	http://www.cazy.org/PULDB/index.php?prot=E7747_14485
11	A0A1C7GWN0	A4V03_00235	Bacteroides caecimuris I48 - 1796613	Predicted PUL 2	http://www.cazy.org/PULDB/index.php?pul=27883
12	D5EY24	PRU_2739	Prevotella ruminicola 23 - 264731	Predicted PUL 23	http://www.cazy.org/PULDB/index.php?prot=PRU_2739
13	A0A077XQF8	BN1088_1431649	Sphingobacterium sp. PM2-P1-29 - 403776	Predicted PUL 28	http://www.cazy.org/PULDB/index.php?prot=BN1088_1431649
14	A0A3G3GQC9	DTQ70_15835	Runella sp. SP2 - 2268026	Predicted PUL 33	http://www.cazy.org/PULDB/index.php?prot=DTQ70_15835
15	D6D0K1	BXY_29300	Bacteroides xylanisolvens XB1A - 657309	Predicted PUL 43	http://www.cazy.org/PULDB/index.php?prot=BXY_29300
16	A0A3B7MJH9	D3H65_11635	Pseudoflavitalea sp. 5GH32-13 - 2315862	Predicted PUL 51	http://www.cazy.org/PULDB/index.php?prot=D3H65_11635
17	B3CER6	BACINT_04197	Bacteroides intestinalis DSM 17393 - 471870	Predicted PUL 56	http://www.cazy.org/PULDB/index.php?pul=85
18	B3CET4	BACINT_04215	Bacteroides intestinalis DSM 17393 - 471870	Predicted PUL 56	http://www.cazy.org/PULDB/index.php?pul=85

19	I3Z663	Belba_2164	Belliella baltica DSM 15883 - 866536	Predicted PUL 7	http://www.cazy.org/PULDB/index.php?prot=Belba_2164
20	A0A1P8E962	BV902_20695	Sphingobacterium sp. B29 - 1933220	Predicted PUL 71	http://www.cazy.org/PULDB/index.php?prot=BV902_20695
21	A0A0P0GBG6	BcellWH2_04301	Bacteroides cellulosilyticus WH2 (new assembly) - 1268240	Predicted PUL 86	http://www.cazy.org/PULDB/index.php?pul=33187
22	D5BGE4	ZPR_0753	Zunongwangia profunda SM-A87 - †655815	Predicted PUL†12	http://www.cazy.org/PULDB/index.php?prot=ZPR_0753
23	A0A1W6E5E5	A6C57_09690	Fibrella sp. ES10-3-2-2 -†1834519	Predicted PUL†17	http://www.cazy.org/PULDB/index.php?pul=34372
24	A0A5C0VGV7	FYC62_01170	Pedobacter sp. CJ43 - †2605747	Predicted PUL†2†	http://www.cazy.org/PULDB/index.php?prot=FYC62_01170
25	I0K8A3	FAES_2347	Fibrella aestuarina BUZ 2 - 1166018	Predicted PUL†20	http://www.cazy.org/PULDB/index.php?prot=FAES_2347
26	A0A2R3MSB2	C3V43_09005	Bacteroides heparinolyticus F0111 - †28113	Predicted PUL†23	http://www.cazy.org/PULDB/index.php?pul=38949
27	D2QDL1	Slin_2106	Spirosoma linguale DSM 74 -†504472	Predicted PUL†23†	http://www.cazy.org/PULDB/index.php?prot=Slin_2106
28	A0A386HQR4	D6B99_10740	Arachidicoccus sp. KIS59-12 -†2341117	Predicted PUL†25	http://www.cazy.org/PULDB/index.php?pul=39964
29	F0S4T7	Pedsa_2561	Pseudopedobacter saltans DSM 12145 - †762903	Predicted PUL†27	http://www.cazy.org/PULDB/index.php?prot=Pedsa_2561
30	A0A5B8VJA9	FSB73_08120	Arachidicoccus ginsenosidivorans Gsoil 809 -†496057	Predicted PUL†28	http://www.cazy.org/PULDB/index.php?pul=47200
31	A0A1H1PLG8	SAMN05216490_0574	Mucilaginibacter mallensis MP1X4 - †652787	Predicted PUL†3	http://www.cazy.org/PULDB/index.php?prot=SAMN05216490_0574
32	L0G017	Echvi_2662	Echinicola vietnamensis DSM 17526 -†926556	Predicted PUL†31	http://www.cazy.org/PULDB/index.php?prot=Echvi_2662
33	G8TR78	Niako_2653	Niastella koreensis GR20-10 -†700598	Predicted PUL†38	http://www.cazy.org/PULDB/index.php?prot=Niako_2653
34	A0A514ZSY2	EXU85_03435	Spirosoma sp. KCTC 42546 -†2520506	Predicted PUL†5†	http://www.cazy.org/PULDB/index.php?prot=EXU85_03435
35	A0A1L7I159	GRFL_0622	Gramella flava JLT2011 - 1229726	Predicted PUL†6†	http://www.cazy.org/PULDB/index.php?prot=GRFL_0622
36	A0A514CP99	FKX85_19365	Echinicola sp. LN3S3 - †2591634	Predicted PUL†63	http://www.cazy.org/PULDB/index.php?prot=FKX85_19365
37	A0A5B8VUC0	FSB73_20480	Arachidicoccus ginsenosidivorans Gsoil 809 -†496057	Predicted PUL†68	http://www.cazy.org/PULDB/index.php?pul=47240
38	X5D7Y3	FH5T_02185	Draconibacterium orientale FH5 - 1168034	Predicted PUL†7†	http://www.cazy.org/PULDB/index.php?prot=FH5T_02185

39	A0A5B9 WRF0	FW415_162 55	Chitinophaga sp. XS-30 -†2604421	Predicted PUL†76†	http://www.cazy.org/PULDB/index.php?prot=FW415_16255
40	E2NGI7	BACCELL_ 03412	Bacteroides cellulosilyticus DSM 14838 - 537012	Predicted PUL†80	http://www.cazy.org/PULDB/index.php?pul=191
41	A0A4U9 W8F5	NCTC11429 _05244	Sphingobacterium thalpophilum NCTC11429 -†259	Predicted PUL†84	http://www.cazy.org/PULDB/index.php?prot=NCTC11429_05244
42	A0A2S1 YTJ1	HYN56_108 60	Flavobacterium crocinum HYN0056 - †2183896	Predicted PUL†9	http://www.cazy.org/PULDB/index.php?prot=HYN56_10860
43	QJD9556 1.1	HH214_065 80	Mucilaginibacter sp. F39-2 - 2728022	Predicted PUL 9	http://www.cazy.org/PULDB/index.php?prot=HH214_06580
44	QCP7244 1.1	FDZ78_075 90	Duncaniella sp. B8 - 2576606	Predicted PUL 10	http://www.cazy.org/PULDB/index.php?prot=FDZ78_07590
45	A0A1W6 E362	A6C57_055 20	n/a	n/a	n/a
46	S0DFK9	BN138_224	n/a	n/a	n/a
47	A0A5M5 ADC2	F3F57_0812 5	n/a	n/a	n/a
48	I0KB42	FAES_3337	n/a	n/a	n/a
49	A0A0H5 Q2Q6	A0A0H5Q2 Q6	n/a	n/a	n/a
50	D9ZDS7	n/a	n/a	n/a	n/a
51	D8L2X7	n/a	n/a	n/a	n/a
52	D5ESF3	PRU_1242	n/a	n/a	n/a
53	P72234	xynC	n/a	n/a	n/a
54	ALJ4715 5.1	n/a	Bacteroides ovatus	n/a	n/a
55	QGT730 69.1	n/a	Bacteroides ovatus	n/a	n/a
56	QDO694 12.1	n/a	Bacteroides intestinalis	n/a	n/a
57	BAV088 92.1	n/a	Filimonas lacunae	n/a	n/a
58	QDW236 02.1	n/a	Pedobacter sp. KBS0701	n/a	n/a
59	QIA0808 8.1	n/a	Draconibacterium sp. M1	n/a	n/a
60	AOW182 04.1	n/a	Polaribacter vadi	n/a	n/a
61	ASB4878 6.1	n/a	Alkalitalea saponilacus	n/a	n/a

n/a, not applicable

Table S3. Relative activity (%) of *Pm*25 and mutants thereof towards polysaccharides.

Substrate	*Pm*25	M 1	M 2
RAX	100 ± 0.89	0.19 ± 0	0.24 ± 0
LVWAX	100 ± 6.57	1.08 ± 0.03	0.76 ± 0.02
Beechwood GX	100 ± 4.67	4.50 ± 0.15	3.08 ± 0.13

Figure legends:

Figure S1. Affinity gel electrophoresis of CBMs in *Pm*25 towards 0.5% (m/v) xyloglucan. M7 *Pm25*ΔCBM4sE546A; M8 CBM4-1; M9 CBM4-2; M10 CBM4-1-CBM4-2;

Figure S2. MST measurement of K_d based on ligand induced effect with X_6. (M7) Inactive catalytic domain without CBMs, *Pm25*ΔCBMs E546A (M8) CBM4-1, (M9) CBM4-2.

Figure S3. Binding assay and affinity gel electrophoresis. (A) Binding of inactive *Pm*25 and its truncated derivatives to wheat bran. BSA was used as negative control. Lane M, molecular mass markers. Lane 1, unbound fraction; lane 2, third wash of the pellet; lane 3, bound fraction. (B) Affinity gel electrophoresis of UNK towards 0.006% (m/v) LVWAX.

Figure S1:

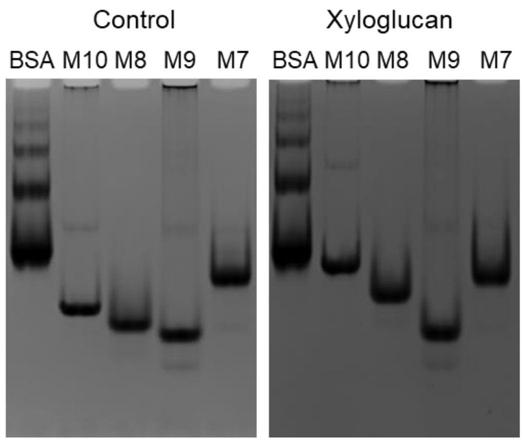

Figure S2:

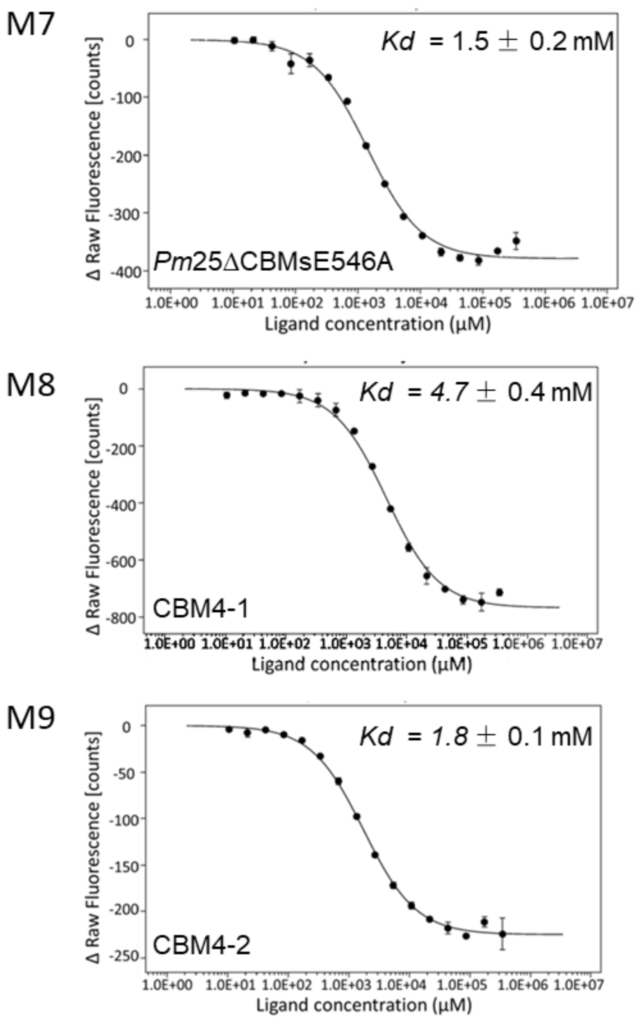
M7
Kd = 1.5 ± 0.2 mM
Pm25ΔCBMsE546A
Δ Raw Fluorescence [counts]
0
-100
-200
-300
-400
1.0E+00 1.0E+01 1.0E+02 1.0E+03 1.0E+04 1.0E+05 1.0E+06 1.0E+07
Ligand concentration (μM)
M8
Kd = 4.7 ± 0.4 mM
CBM4-1
Δ Raw Fluorescence [counts]
0
-200
-400
-600
-800
1.0E+00 1.0E+01 1.0E+02 1.0E+03 1.0E+04 1.0E+05 1.0E+06 1.0E+07
Ligand concentration (μM)
M9
Kd = 1.8 ± 0.1 mM
CBM4-2
Δ Raw Fluorescence [counts]
0
-50
-100
-150
-200
-250
1.0E+00 1.0E+01 1.0E+02 1.0E+03 1.0E+04 1.0E+05 1.0E+06 1.0E+07
Ligand concentration (μM)

Figure S3:

A

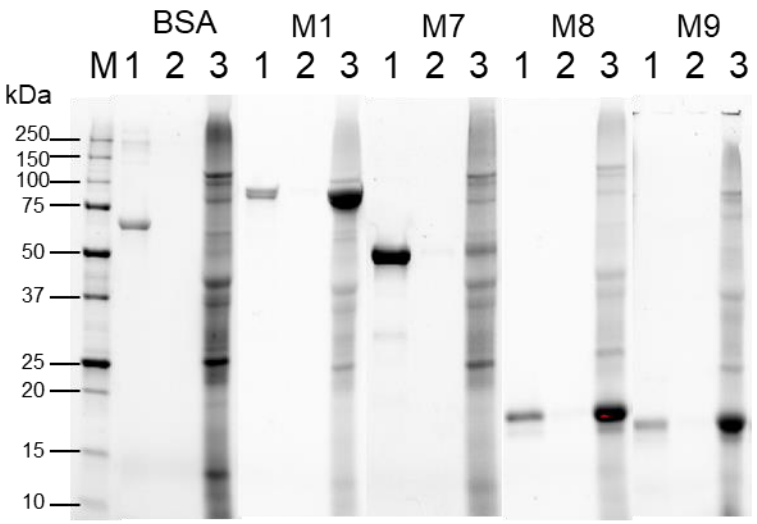
BSA
M1
M7
M8
M9
M 1 2 3 1 2 3 1 2 3 1 2 3 1 2 3
kDa
250
150
100
75
50
37
25
20
15
10

B

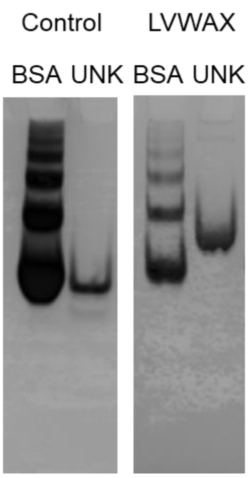
Control
LVWAX
BSA UNK BSA UNK
